# Supplementary material for: miR-93 regulates Msk2-mediated chromatin remodelling in diabetic nephropathy
Source: Nat Commun. 2016 Jun 28;7:12076. doi: 10.1038/ncomms12076 (PMC4931323; doi:10.1038/ncomms12076)
Supplement: Supplementary Information — Supplementary Figures 1-10 and Supplementary Tables 1 & 2. [file ncomms12076-s1.pdf]

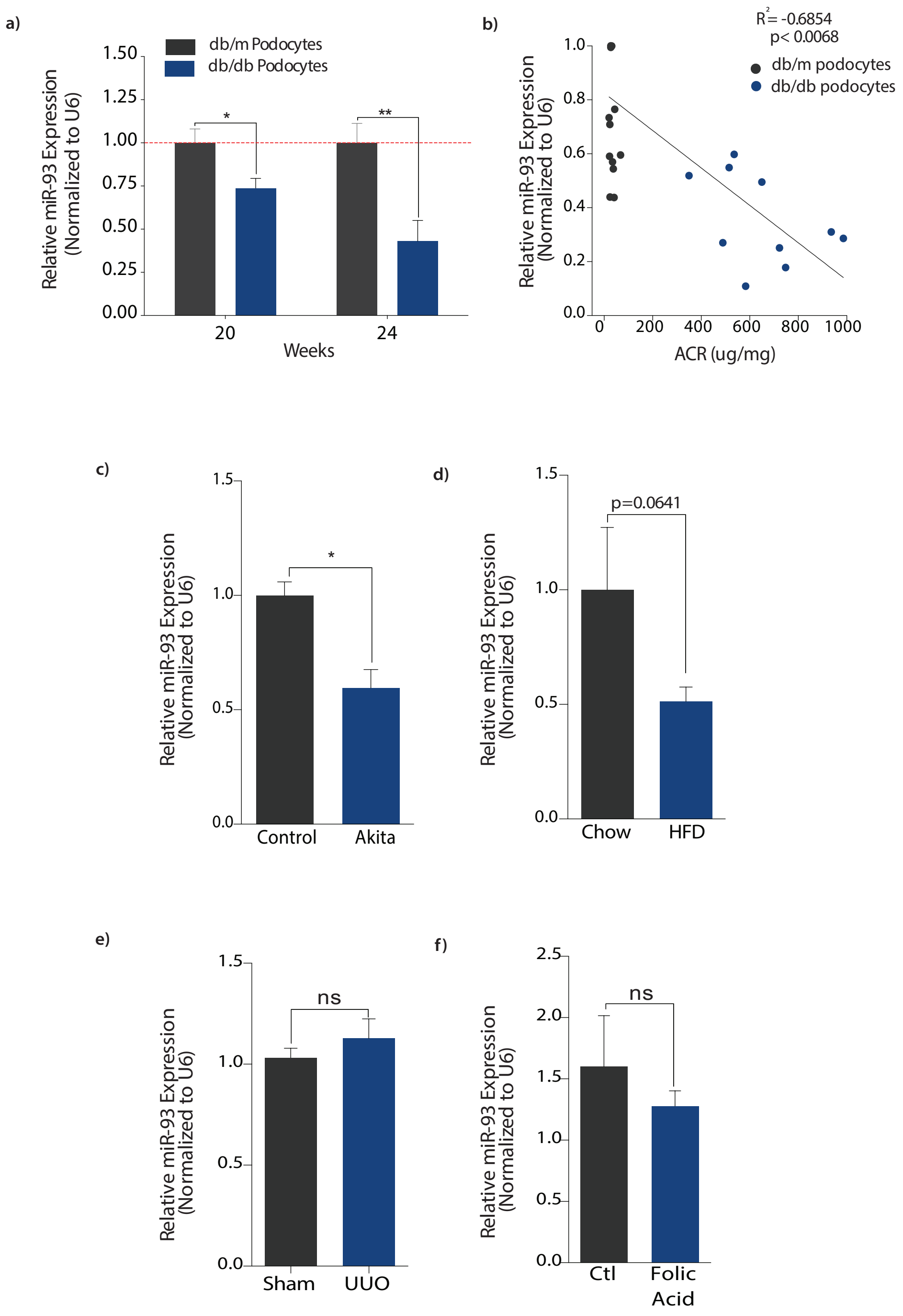

Supplementary Figure 1: miR-93 levels in several models of kidney injury

a) Gene expression analysis of miR-93 expression in podocytes isolated from db/m and db/db mice at two different time points (n=5/group at each time point). All values were normalized to U6 snRNA internal control. b) Linear regression analysis of relative miR-93 expression values from (a) plotted against ACR from 24-week-old db/m or db/db. (n=8-12 mice/group (a). c) Analysis of miR-93 levels in podocytes isolated from non-diabetic (n=4) and Akita (n=7) mice on the DBA2J background. d) Gene expression analysis of miR-93 levels in podocytes isolated from mice on the C57Bl6J background placed on either a Chow Diet (n=2) or High Fat Diet (HFD, n=4). e) Gene expression analysis of miR-93 levels in kidney cortex from mice subjected to unilateral ureteral obstruction (UUO) for 10 days (n=4) or sham operated control kidneys (n=4) f) qPCR analysis from kidney cortex from mice administered folic acid (50mg/kg) or vehicle control for 21 days (n=3 mice/group). All expression values are normalized to U6 snRNA internal controls. Data are expressed as mean ± s.e.m, ns: no significance, * P<0.05, **P<0.0. . Student's t-test was employed for comparisons between two groups.

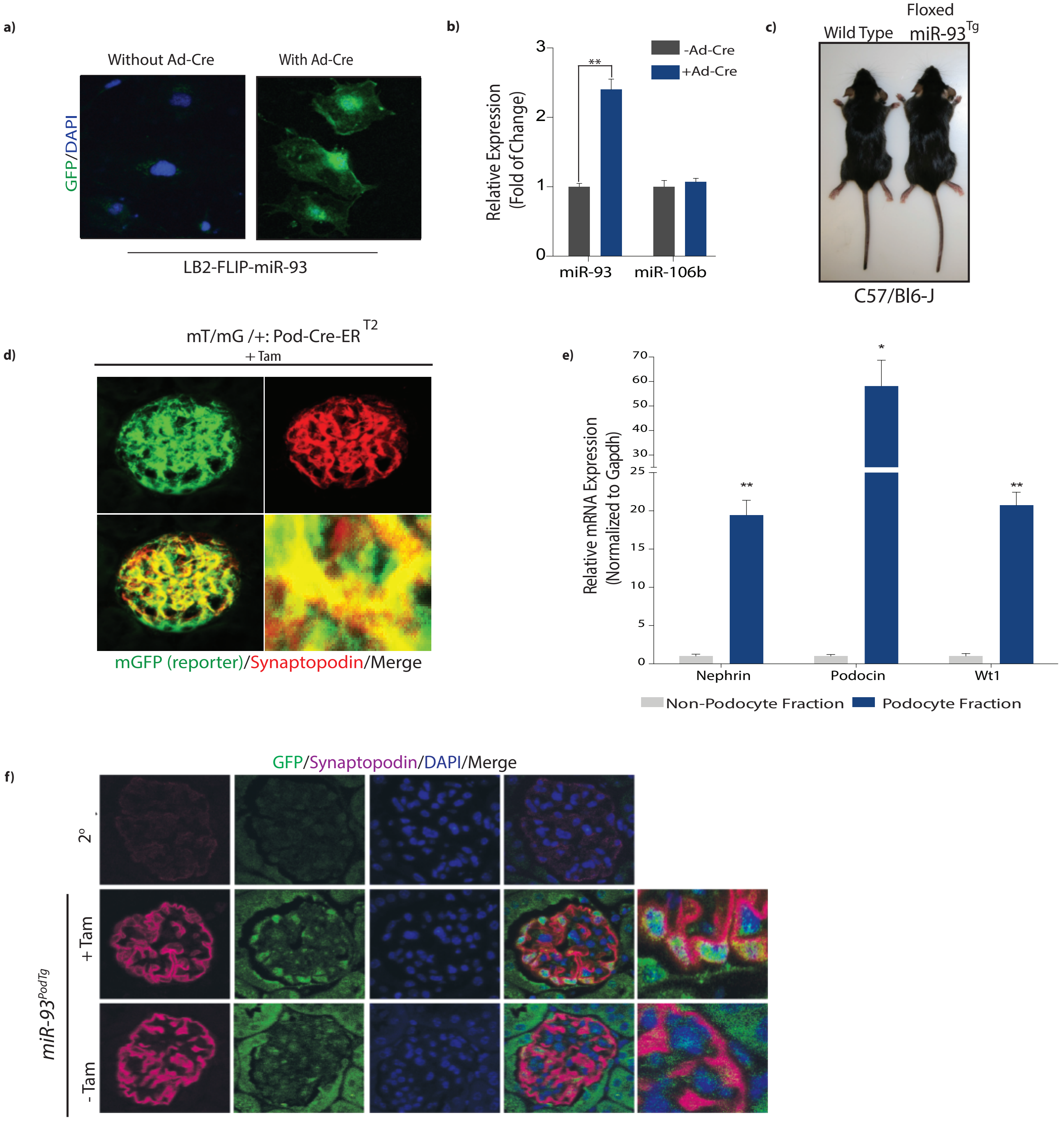

Supplementary Figure 2: Generation and characterization of floxed miR-93 transgenic mice
a) Immunofluorescence analysis of stably transduced LB2-FLIP-miR-93 floxed podocytes receiving adenovirus-Cre to induce recombination and GFP-miR-93 expression. b) qPCR analysis of miR-93 and miR-106b expression (negative control) in podocytes from (a). c) Representative wild-type and miR-93Tg mice prior to crossing with the inducible, podocyte specific Cre. d) Representative confocal micrographs of kidney sections from tamoxifen induced R26mTmG;Pod-CreERT2 mice stained with synaptopodin. mGFP signal (Green) overlaps with Synaptopodin signal (Red) to demonstrate podocyte specific activity of the inducible Cre recombinase. e) In order to validate our podocyte enrichment protocol we performed qPCR analysis of podocyte specific mRNAs from podocyte (+) versus podocyte (–) cell fractions. Data are from cell fractions isolated from five different animals. f) To confirm the podocyte specific induction and flipping of our transgenic construct we performed immunofluorescence analysis against transgenic GFP and Synapotpodin. Representative confocal micrographs of kidney sections from -/+ tamoxifen induced miR-93PodTg mice stained for GFP and synaptopodin to demonstrate podocyte specific induction of the miR-93 floxed transgenic construct. Scale bars denote 50µm. Insets are meant to demonstrate podocyte localization. Data are expressed as mean ± s.e.m. *P<0.05, **P<0.01. . Student's t-test was employed for comparisons between two groups; one-way analysis of variance with Tukey's post test for multiple comparisons was used for groups of three or more.

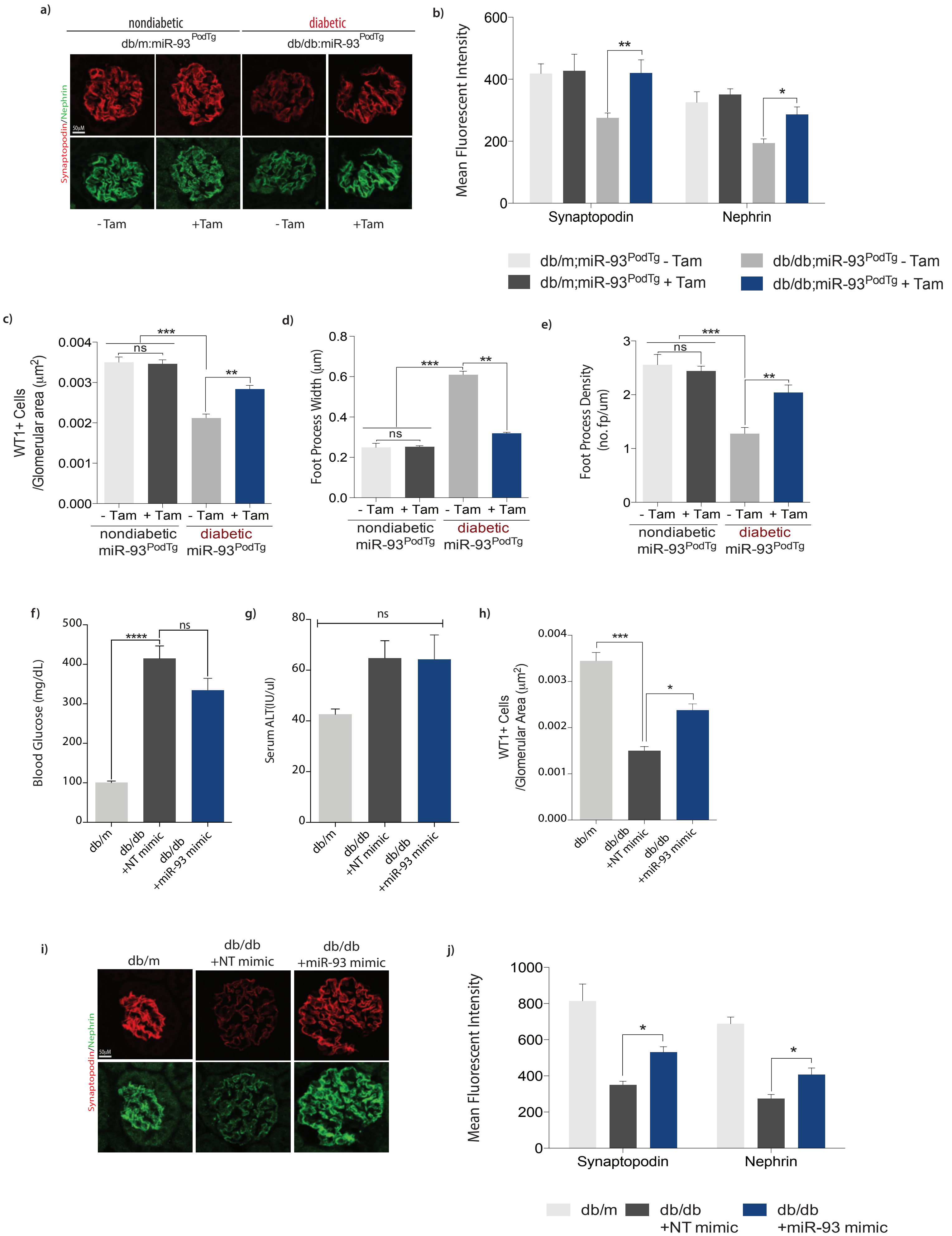
a)
nondiabetic
diabetic
db/m:miR-93 PodTg
db/db:miR-93 PodTg
Synaptopodin/Nephrin
50uM
- Tam
+Tam
- Tam
+Tam
b)
Mean Fluorescent Intensity
Synaptopodin
Nephrin
db/m;miR-93PodTg - Tam
db/m;miR-93PodTg + Tam
db/db;miR-93PodTg - Tam
db/db;miR-93PodTg + Tam
c)
WT1+ Cells /Glomerular area (μm2)
ns
- Tam + Tam - Tam + Tam
nondiabetic miR-93PodTg
diabetic miR-93PodTg
d)
Foot Process Width (μm)
e)
Foot Process Density (no. fp/um)
f)
Blood Glucose (mg/dL)
g)
Serum ALT(IU/ul)
h)
WT1+ Cells /Glomerular Area (μm2)
db/m
db/db +NT mimic
db/db +miR-93 mimic
i)
db/m
db/db +NT mimic
db/db +miR-93 mimic
Synaptopodin/Nephrin
50uM
j)
Mean Fluorescent Intensity
Synaptopodin
Nephrin
db/m
db/db +NT mimic
db/db +miR-93 mimic

Supplementary Figure 3: miR-93 exerts a renoprotective effect in diabetic nephropathy

a) Representative immunofluorescence images of kidney sections from control db/m;miR-93PodTg (n=3), tamoxifen induced db/m;miR-93PodTg (n=3), control db/db;miR-93PodTg (n=5) and tamoxifen induced db/db;miR-93PodTg (n=5) animals stained with antibodies against synaptopodin and nephrin. b) Quantification of synaptopodin and nephrin glomerular mean fluorescence intensity from the groups in (a). c) Quantification of WT1+ cells from kidney sections from groups in (a). Total number of WT1+ cells per glomerular section was divided by glomerular area (μm2). d-e) Quantification of foot process width (d) and foot process density (e) calculated from TEM micrographs from groups in (a). f-g) Fasting blood glucose (f) and serum alanine aminotransferase (ALT) (g) levels in db/m (n=8) and db/db mice administered miR-93 mimics (n=8) or Nontargeting (NT) mimics (n=8). h) Quantification of WT1+ cells from kidney sections from mice in (f) stained with an antibody against WT1, quantified as before. i) Representative immunofluorescence images of kidney sections from control db/m (n=3), dbdb+miR-93 mimics (n=4) and db/db+NT mimics (n=4) animals stained with antibodies against synaptopodin and nephrin. Sections were counterstained with DAPI. Scale bars denote 50μm j) Quantification of synaptopodin and nephrin glomerular mean fluorescence intensity from the groups in (i). Data are expressed as mean ± s.e.m, ns: no significance, * $P<0.05$, ** $P<0.01$, *** $P<0.001$, ****$P<0.0001$. one-way analysis of variance with Tukey's post test for multiple comparisons was used for groups of three or more.

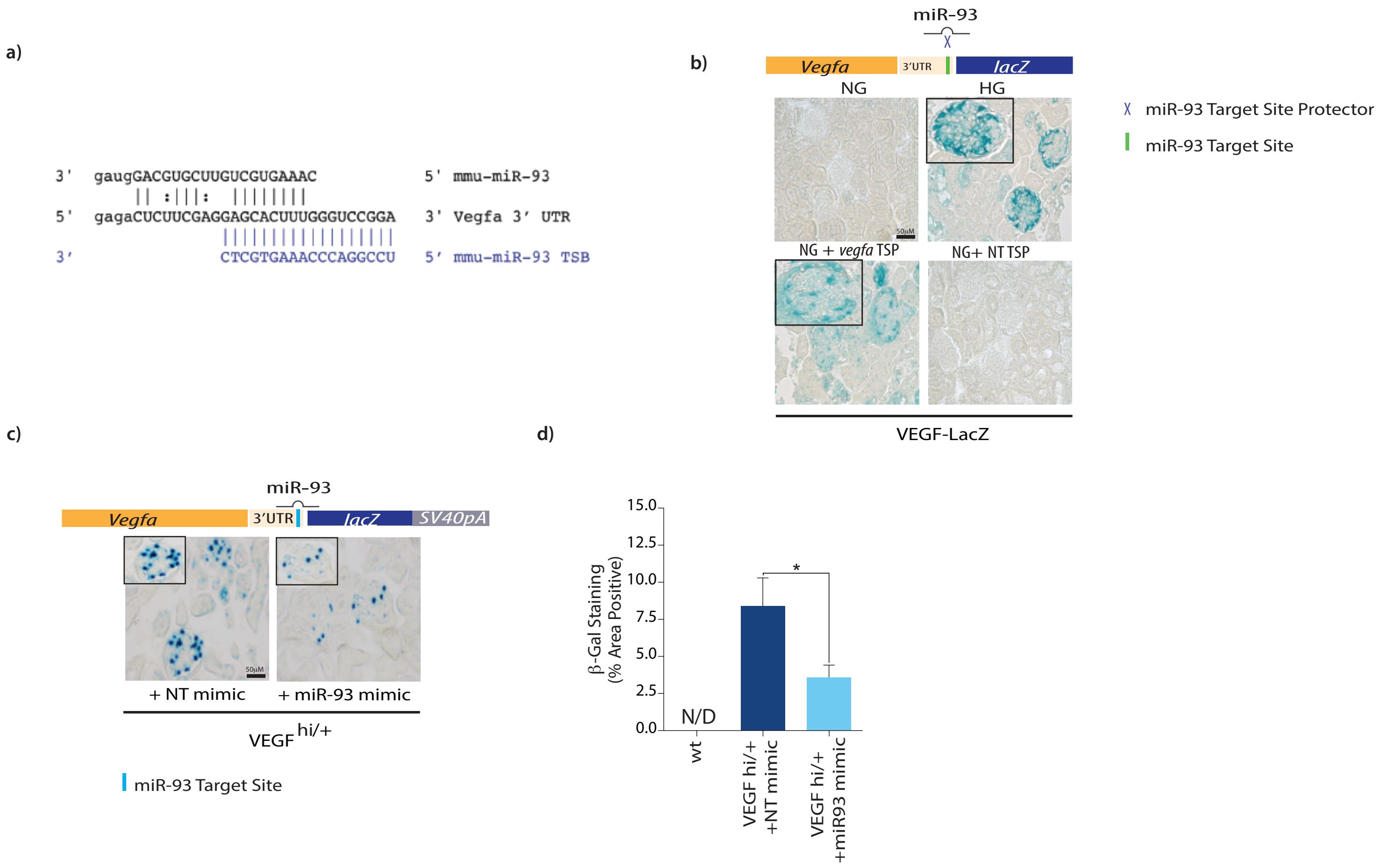

Supplementary Figure 4: miR-93 inhibits VEGF Expression in vivo

a) To test the role of VEGF as a target of miR-93 in vivo, we designed a miR-93 Target Site Protector (TSP), to block miR-93 binding to its cognate seed sequence in the VEGF 3'-UTR. The wild-type VEGF 3'-UTR, the miR-93 TSP and the mature miR-93 sequence are depicted in (a), a nontargeting (NT) TSP was used as a negative control. b) Kidney cortices from VEGF-LacZ reporter mice (Miquerol et al., Dev. Biol, 1999) with normal expression levels of VEGF were cultured in normal glucose (NG, 5mM) with either a NT TSP or the miR-93 TSP. We found a marked increase in VEGF expression in miR-93-TSP-treated kidney cortices in NG conditions as determined by β-galactosidase activity. Increased VEGF expression in HG (25mM) conditions served as a positive control. We did not observe any staining in NG or NT-TSP cultured cortices; suggesting endogenous miR-93 exerts tight control over VEGF expression in vivo. Scale bars denote 50 mm; n=3 mice/group c) To show miR-93 is able to attenuate VEGF expression in a system with constitutive VEGF overexpression, we utilized the VEGF-Hi Reporter mice (Miquerol et al., Development, 2000). The VEGF-Hi reporter mice exhibit constitutive overexpression of VEGF due to increased mRNA stability, and retain the conserved miR-93 binding site within the remnant 3'-UTR. Depicted is the schematic of the VEGF-Hi-LacZ reporter allele in the top panel. VEGF-Hi mice received miR-93 mimics via i.p. injection, and expression of the VEGF reporter allele was assessed by visualizing β-galactosidase activity. As expected, VEGF expression was markedly elevated within podocytes and in tubular epithelial cells in VEGF-Hi mice receiving the NT mimic. However, VEGF-Hi mice that received miR-93 mimics displayed significantly reduced VEGF expression. d) Quantification of percent area positive for β-gal staining from (c). Taken together, these observations suggest that VEGF is a strong in vivo target of miR-93. Scale bars denote 50µm; n=4 mice/group Data expressed as mean± s.e.m. ns: no significance, *P<0.05, **P<0.01, ***P<0.001. one-way analysis of variance with Tukey's post test for multiple comparisons was used for groups of three or more.

a)

<table>
<tr><th>miRNA Name</th><th>miRNA Seed</th><th>Seed Model</th><th>Length of Complementarity</th><th>Complementarity</th><th>Viterbi P-Value</th></tr>
<tr><td>miR-20a, miR-106b</td><td>GCACTTTA</td><td>8mer</td><td>8</td><td>GCACUUUA
||||||||
CGUGAAAU</td><td>1.50E-05</td></tr>
<tr><td>miR-17, miR-20b, miR-93, miR-106a</td><td>GCACTTT</td><td>7mer</td><td>7</td><td>GCACUUUA
|||||||
CGUGAAA-</td><td>6.10E-05</td></tr>
<tr><td>miR-20a, miR-106b</td><td>GCACTT</td><td>6mer</td><td>6</td><td>GCACUU
||||||
CGUGAA</td><td>0.000244</td></tr>
<tr><td>miR-17, miR-20b, miR-93, miR-106a</td><td>GCACTT</td><td>6mer</td><td>6</td><td>GCACUU
||||||
CGUGAA</td><td>0.000244</td></tr>
<tr><td>miR-93*</td><td>TCAGCA</td><td>6mer</td><td>1</td><td>GCACUU-----
|
-----AGUCGU</td><td>0.384277</td></tr>
</table>

b)

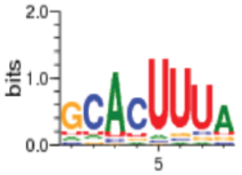

c)

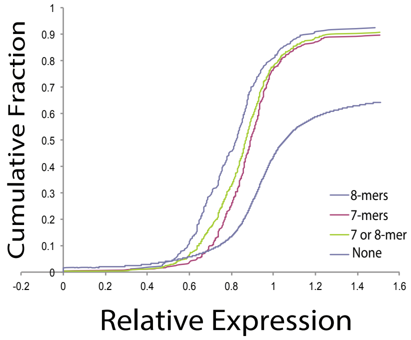

d)

	Avg. FPKM (n=2)						
	NT_mimic	miR-93_mimic	log2_fold_change	test_stat	p_value	q_value	significant
Rps6ka4 (Msk2)	14.7236	9.6805	-0.604982	-5.96614	2.43E-09	1.70E-07	yes

e)

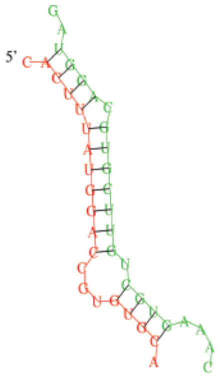

mfe: -17.2 kcal/mol

```
405:5' acACC--CAUCAAUGUatCcaca 3'  Msk2 3'UTR Mut
          |||   ||: ||::    |
       3' gaUGGACGUGCUUGUCGUGAAAc 5'  mmu-miR-93
          |||   ||: ||:: ||||||
405:5' acACC--CAUCAAUGUCACUUUa 3'  Msk2 3'UTR WT
```

f)

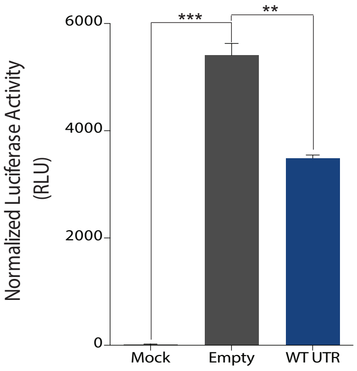

g)

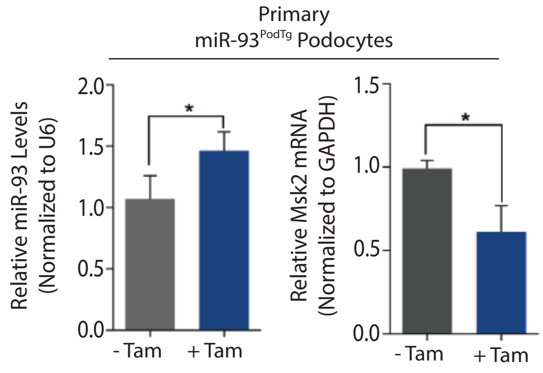

h)

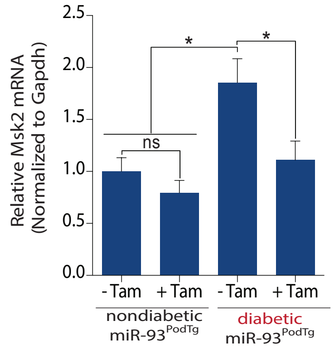

Supplementary Figure 5: miR-93 targets are enriched in RNA-Seq analysis

a) microRNA seed enrichment analysis in significantly downregulated genes following miR-93 overexpression. b) Meme analysis of significantly enriched microRNA seed motif. c) Cumulative distribution of genes with different number of seeds. d) Statistical analysis and average fragments per kilobase of transcript per Million mapped reads (FPKM) values from RNA-Seq analysis of immortalized mouse immortalized podocytes transfected with either NT mimics (n=2) or miR-93 mimics (n=2). e) left, mRNA-miRNA secondary structure analysis between miR-93 and mouse Msk2 3'-UTR Right, Sequence alignment of Msk2 wild type (WT) 3'UTR and mutant (Mut). Nucleotides in red denote the mutated miR-93 binding site. f) Inclusion of the full length WT Msk2 3'-UTR attenuates luciferase activity in HEK293T cells compared to empty vector controls. g) qPCR analysis for miR-93 and Msk2 expression in primary miR-93PodTg podocytes cultured with 1μM 4-OH tamoxifen compared to non-treated controls (n=3/group). microRNA values were normalized to U6 snRNA internal controls. h) qPCR analysis of Msk2 mRNA in kidney cortices from tamoxifen-induced non-diabetic and diabetic miR-93PodTg mice compared with non-induced controls (n=4 mice/group). mRNA expression values normalized to Gapdh internal controls. Data are expressed as mean ± s.e.m. ns: no significance, *P<0.05, **P<0.01, ***P<0.001. . Student's t-test was employed for comparisons between two groups; one-way analysis of variance with Tukey's post test for multiple comparisons was used for groups of three or more.

a)

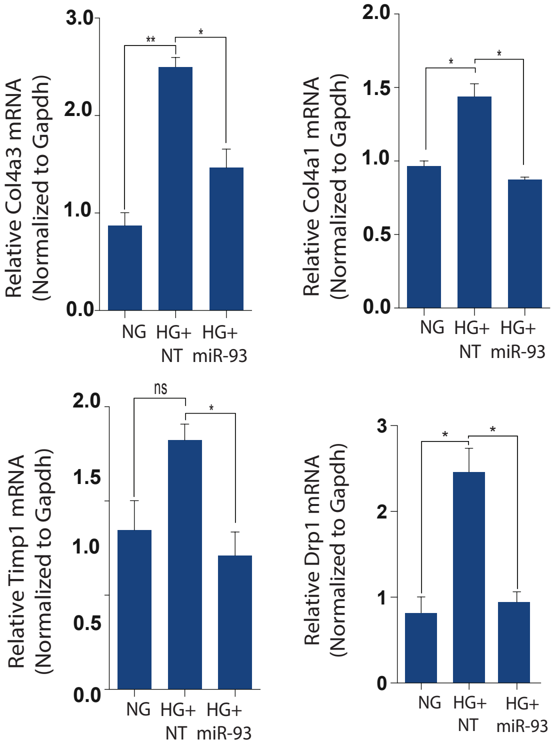

Supplementary Figure 6: miR-93 overexpression reverses HG-induced mRNA levels of several DN related genes in podocytes

a) Gene expression analysis of several well known pathogenic genes within DN, including Col4a3, Col4a1, Timp1, and Dnm1l (Drp1) from podocytes cultured with HG + miR-93 mimics compared with podocytes cultured with HG + NT control mimics. Data are expressed as mean ± s.e.m. ns: no significance, *P<0.05, **P<0.01. one-way analysis of variance with Tukey's post test for multiple comparisons was used for groups of three or more.

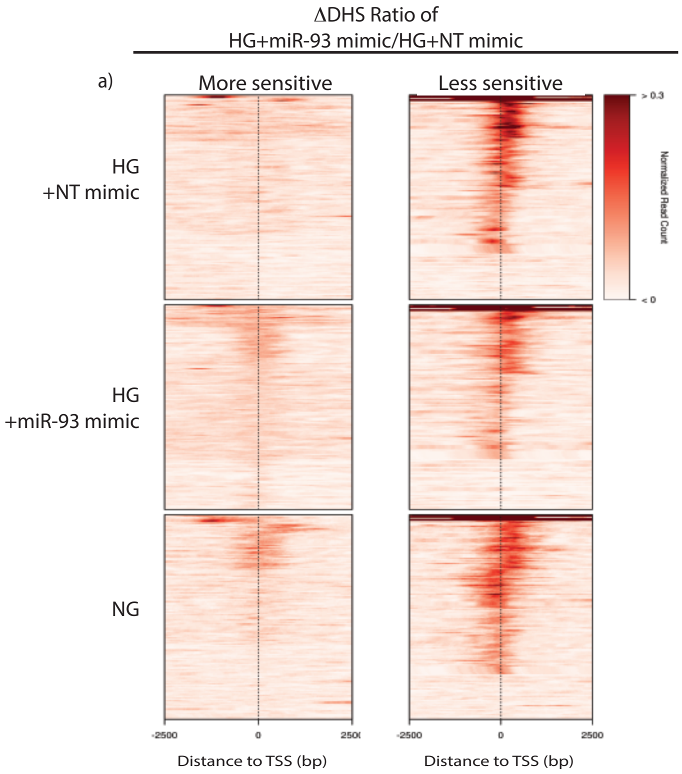

ΔDHS Ratio of
HG+miR-93 mimic/HG+NT mimic

b)

More sensitive regions (log$_2$FC > 0.75)

	Gene Set Name	k/K	p-value	FDR q-value
1	MEMBRANE	0.0843	1.48E-39	3.74E-36
2	PLASMA_MEMBRANE	0.0947	5.72E-37	7.23E-34
3	PLASMA_MEMBRANE_PART	0.0976	3.71E-32	1.57E-29
4	SIGNAL_TRANSDUCTION	0.0832	2.04E-31	7.37E-29
5	INTRINSIC_TO_MEMBRANE	0.0875	3.08E-29	8.67E-27
6	SYSTEM_PROCESS	0.1279	7.83E-28	1.98E-25
7	INTEGRAL_TO_MEMBRANE	0.0857	1.77E-27	4.08E-25
8	INTRINSIC_TO_PLASMA_MEMBRANE	0.0938	2.38E-25	5.03E-23
9	PROTEIN_METABOLIC_PROCESS	0.0772	7.26E-20	1.15E-17
10	CELLULAR_PROTEIN_METABOLIC_PROCESS	0.077	5.10E-18	5.87E-16
11	CELL_CELL_SIGNALING	0.1015	5.53E-13	3.68E-11
12	ESTABLISHMENT_OF_LOCALIZATION	0.0736	7.74E-13	4.90E-11

Less sensitive regions (log$_2$FC > 0.75)

	Gene Set Name	k/K	p-value	FDR q-value
1	REACTOME_TRANSCRIPTION	0.1619	1.37E-24	3.82E-22
2	REACTOME_DEPOSITION_OF_NEW_CENPA_CONTAINING_N UCLEOSOMES_AT_THE_CENTROMERE	0.2969	1.22E-19	2.10E-17
3	BIOPOLYMER_METABOLIC_PROCESS	0.0481	1.46E-19	2.32E-17
4	REACTOME_CHROMOSOME_MAINTENANCE	0.1885	1.30E-18	1.82E-16
5	SIGNAL_TRANSDUCTION	0.0447	5.15E-16	6.03E-14
6	RNA_METABOLIC_PROCESS	0.0595	5.94E-16	6.62E-14
7	NUCLEOBASENUCLEOSIDENUCLEOTIDE_AND_NUCLEIC_AC ID_METABOLIC_PROCESS	0.0498	7.06E-16	7.49E-14
8	RNA_BIOSYNTHETIC_PROCESS	0.0643	2.07E-14	2.10E-12
9	TRANSCRIPTION_DNA_DEPENDENT	0.0629	8.67E-14	8.05E-12
10	TRANSCRIPTION_FROM_RNA_POLYMERASE_II_PROMOTER	0.07	1.63E-12	1.34E-10
11	CELLULAR_MACROMOLECULE_METABOLIC_PROCESS	0.0433	1.19E-10	9.13E-09
12	PROTEIN_METABOLIC_PROCESS	0.0414	2.33E-10	1.73E-08

Supplementary Figure 7: DNase-Seq aggregate line plots and GSEA analysis
a) Aggregate line plots demonstrating the cumulative distribution of reads surrounding TSSs at sites which either gain (left panel) or lose (right panel) hypersensitivity. b) Gene Set Enrichment GSEA analysis of genes near TSSs which gain or lose hypersensitivity in podocytes cultured in HG + miR-93 mimics compared to podocytes cultured in HG+NT mimics. Genes with ± log2 0.75 FC were used in the GSEA analysis. Gene sets highlighted in red, suggest miR-93 overexpression results in loss of hypersensitivity near genes that are important for gene transcription.

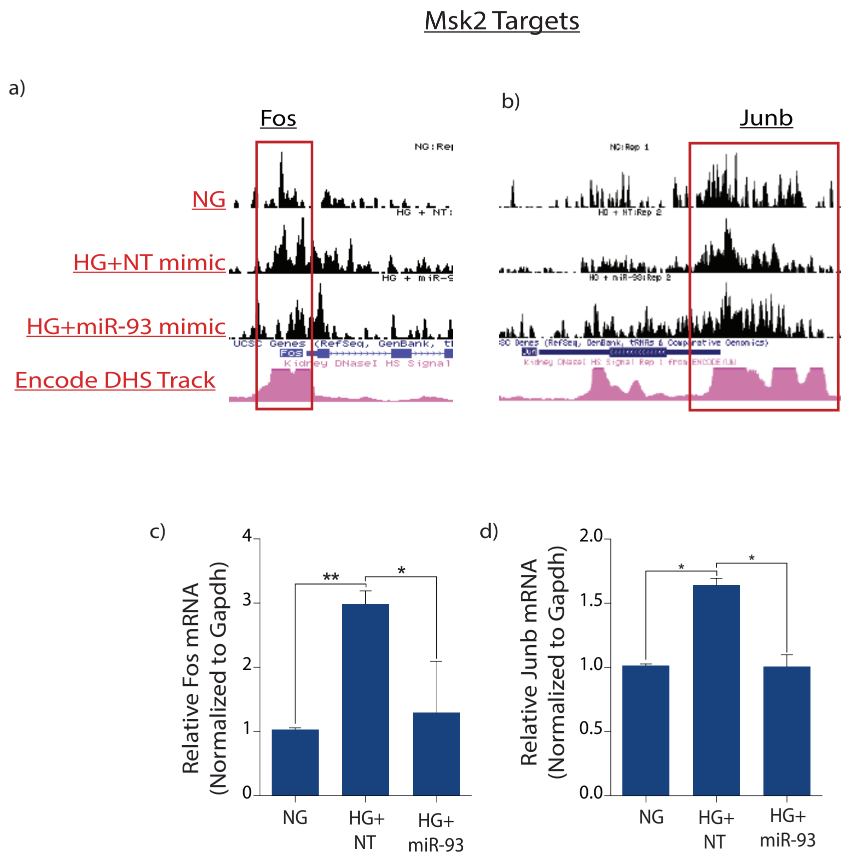

Supplementary Figure 8: DNase-Seq and RNA expression analysis at Msk2 targets
a-b) Selected signal tracks from DNase-hypersensitivity assays from NG, HG+NT, HG+miR-93 treated podocytes. Selected genes include well-known Msk2 target genes, Fos and Jun. As reference, DHS signal tracks from the Encode project are depicted in pink c-d) Gene expression analysis of Fos and Jun from RNA isolated from podocytes used for DNase-hypersensitivity assays. mRNA expression values normalized to Gapdh internal controls. Data are expressed as mean± s.e.m. *P <0.05, **P-<0.01. one-way analysis of variance with Tukey's post test for multiple comparisons was used for groups of three or more.

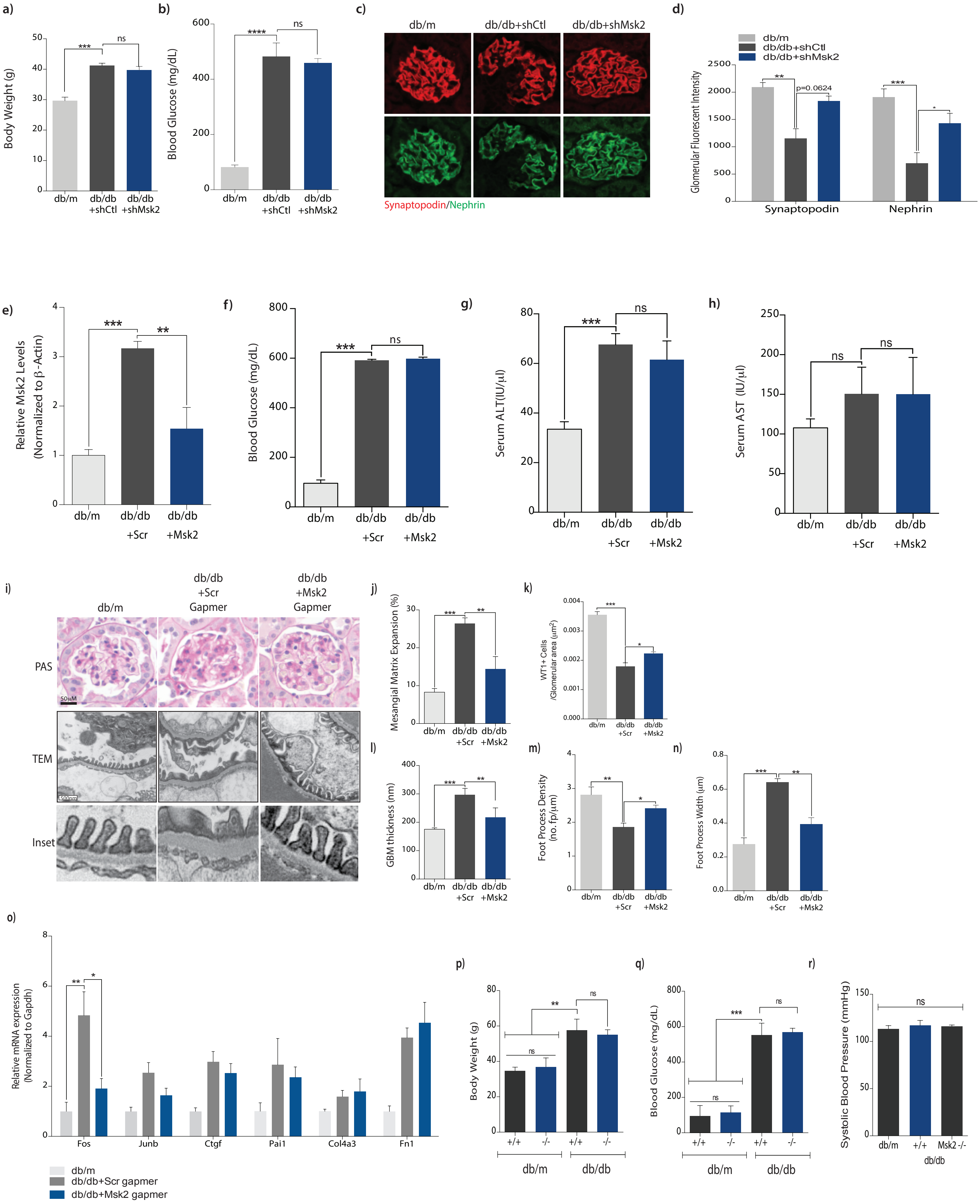

a)
Body Weight (g)
db/m
db/db +shCtl
db/db +shMsk2
b)
Blood Glucose (mg/dL)
c)
db/m
db/db+shCtl
db/db+shMsk2
Synaptopodin/Nephrin
d)
db/m
db/db+shCtl
db/db+shMsk2
Glomerular Fluorescent Intensity
p=0.0624
Synaptopodin
Nephrin
e)
Relative Msk2 Levels (Normalized to β-Actin)
db/m
db/db +Scr
db/db +Msk2
f)
Blood Glucose (mg/dL)
g)
Serum ALT(IU/μl)
h)
Serum AST (IU/μl)
i)
db/m
db/db +Scr Gapmer
db/db +Msk2 Gapmer
PAS
TEM
Inset
j)
Mesangial Matrix Expansion (%)
k)
WT1+ Cells /Glomerular area (μm²)
l)
GBM thickness (nm)
m)
Foot Process Density (no.fp/μm)
n)
Foot Process Width (μm)
o)
Relative mRNA expression (Normalized to Gapdh)
Fos
Junb
Ctgf
Pai1
Col4a3
Fn1
db/m
db/db+Scr gapmer
db/db+Msk2 gapmer
p)
Body Weight (g)
+/+
-/-
db/m
db/db
q)
Blood Glucose (mg/dL)
r)
Systolic Blood Pressure (mmHg)
Msk2 -/-

Supplementary Figure 9: Msk2 depletion in vivo attenuates features of DN
a-b) Body weight and blood glucose levels from db/m (n=4), db/db+shCtr (n=3) and db/db+shMsk2 (n=5) c) Representative immunofluorescence micrographs of kidneys from mice in (a) stained with anti-synaptopodin or anti-nephrin antibodies. Sections were counterstained with DAPI. Scale bars denote 50 μM. d) Quantification of synaptopodin and nephrin glomerular mean fluorescence intensity from the groups in (a). e) Densitometric analysis of western blots from Fig. 6m demonstrating depletion of Msk2 f-h) Fasting blood glucose (f), serum Aspartate Aminotransferase (AST) (g), and serum Alanine Aminotransferase (ALT) levels (h) in db/m (n=4), db/db+Scramble (Scr) gapmer (n=5) and db/db+Msk2 Gapmer (n=5). i) Representative images of PAS Staining and TEM micrographs. Scale bars denote 50 μM (first row) 0.5 μM (second row). Merged inset highlights podocyte specific H3S10P. j-n) Quantification of mesangial matrix expansion (j), WT1+ cells (k), GBM Thickness (l), Foot process density (m) and foot process width (n) in gapmer treated mice and controls. o) Gene expression analysis of RNA isolated from from kidneys of db/m (n=3), db/db+Msk2 (n=4) and db/db+Scr (n=4) gapmers. Expression values normalized to Gapdh internal controls. p-r) body weight (p), fasting blood glucose (q) measurements from db/m (n=3), db/m;Msk2-/- (n=3), db/db (n=3) and db/db;Msk2-/- (n=5). r) Systolic blood pressure measurements, from db/m, db/db and db/db;Msk2-/- mice. n=2 mice/group was utilized. Data are expressed as mean± s.e.m. *P <0.05, **P<0.01, ***P<0.001, ****P<0.0001.

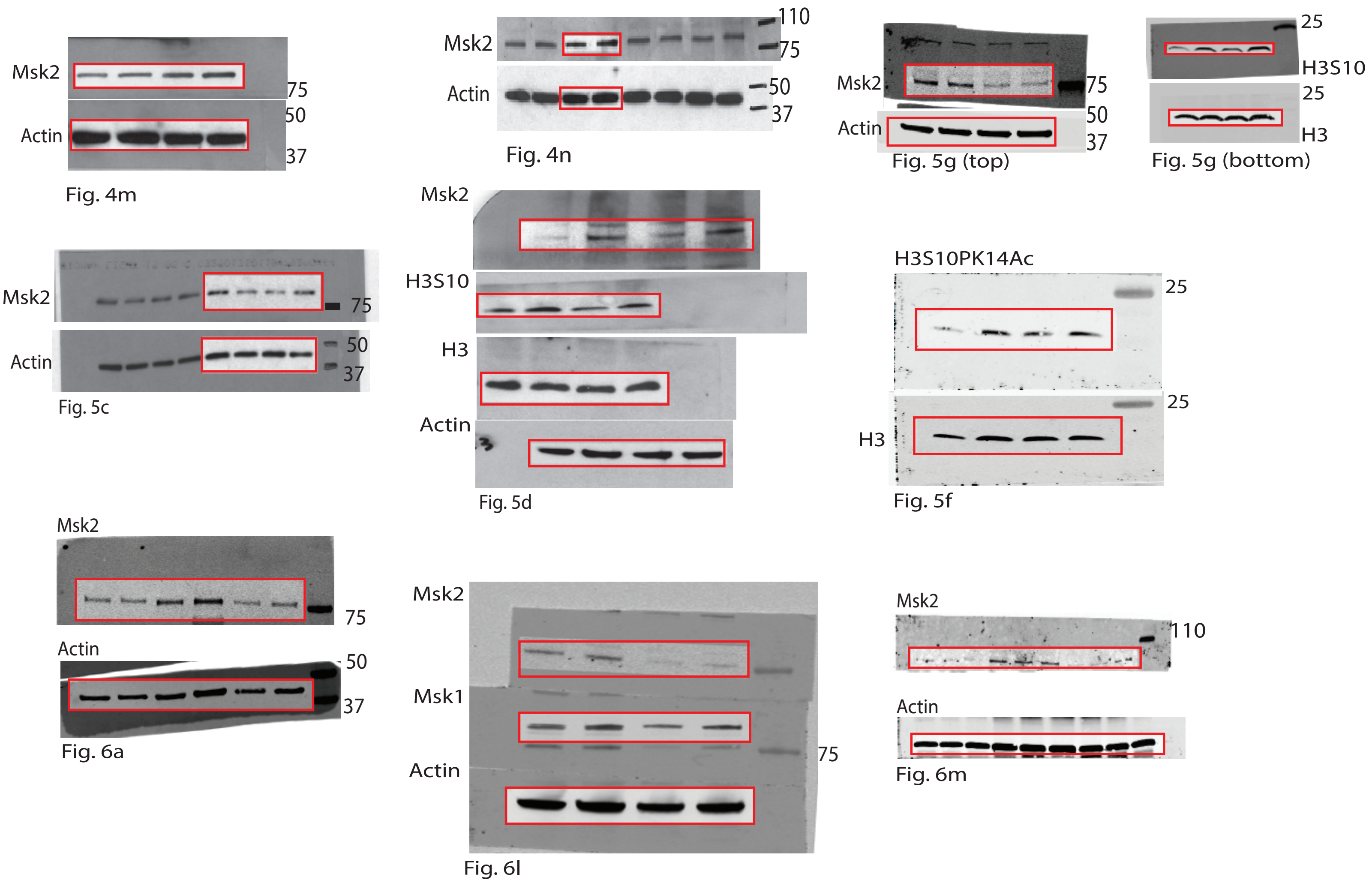

Supplementary Figure 10: Original Western Blots. Red boxes highlight area presented in corresponding figures.

Supplementary Table 1	Control (n=8)	DN (n=7)	P-Value
**Age (Yrs.)**	57.5±11.5	61.1±10.6	ns
**Sex (M/F)**	6/2	4/3	
**Macroalbuminura (>300 mg/dl)**	0	3/6	
**Serum Creatinine (mg/dL)**	0.99±0.21	2.57±1.14	<0.01
**eGFR (ml/min/1.73m2)**	79.81±15.52	31.07±18.10	<.001

Supplementary Table 1: Characteristics of control subjects and patients with DN. Data are expressed as mean± s.e.m. ns: no significance.

qPCR (cDNA)	Gene:	Forward:	Reverse:
Mouse	Gapdh	GCCTGGAGAAACCTGCCAA	CGAAGGTGGAAGAGTGGGAG
	Msk2	GGTGAGCGTGGAGAACTTCG	GAAGGCGTAGTGCAGTGTGA
	Col4a3	AGAGGGGACGAGGGCGGAAC	TCCCCGGCGGGACACAGATT
	Col4a1	TTCTCTTCTGCAACATCAAC	GAATCTGAATGGTCTGACTG
	Ctgf	GAGGAAAACATTAAGAAGGGC	AGAAAGCTCAAACTTGACAG
	Fn1	CCTATAGGATTGGAGACACG	GTTGGTAAATAGCTGTTCGG
	Tgfb1	GGATACCAACTATTGCTTCAG	TGTCCAGGCTCCAAATATAG
	Timp1	CATCCTCTTGTTGCTATCAC	CATGAATTTAGCCCTTATGACC
	Serpine1	AGCAACAAGTTCAACTACAC	CTTCCATTGTCTGATGAGTTC
	Rock1	GACTGGGGACAGTTTTGAGAC	GGGCATCCAATCCATCCAGC
	Dnm1l	GATTCAATCCGTGATGAGTATG	TAAGTAACCTATTCAGGGTCC
	Fos	GAAGGGAACGGAATAAGATG	CATCTTCAAGTTGATCTGTCTC
	Junb	GAACAGCCTTTCTATCACG	GTTTCAGGAGTTTGTAGTCG
	Wt1	GTAAAACAAGTGAAAAGCC	TCAGATTTGGAAGCAGTTTG
		**TaqMan Assay ID**	
Mouse/Human	mmu-mir-93	1090	
	U6 snRNA	1973	
		**Forward:**	**Reverse:**
Genotyping Primers	GFP/miR-93	TGCTCAGGTAGTGGTTGTCG	CACATGAAGCAGCACGAC
	Puro	GTCACCGAGCTGCAAGAACT	CAGGAGGCCTTCCATCTGT
	Thy1.1	CGCTCTCCTGCTCTCAGTCT	GCACGTGCTTCCTCTTCTCT
	Pod-iCre	TCAACATGCTGCACAGGAGAT	ACCATAGATCAGGCGGTGGGT
	VEGF-Wt	ATGTGACAAGCCAAGGCGGTG	-
	VEGF-exon8	TGGCGATTTAGCAGCCAGATA	-
	VEGF-LacZ	-	GGTAGGGGTTTTTCACAGAC
	Msk2KO	CGTTGGCTACCCGTAATATTGCTGAAGAGC	
	Msk2WT	AAGATCTTCAGGGCATCTCTTTATCCTACG	
	Msk2KO-WT	TTGTGCTCCCCATGCTGCAGCCCGGCCTTC	
		**Forward:**	**Reverse:**
Subcloning Primers	mMsk2_3UTR	CTCGAGCTCCCGCCACTGTGAC	GAATTCTGCAGCACTGGGTGGAG
	mMsk2 WT_1	CTCTGCTGCCTCCTGGCTTCT	CGAGGCGGATCACAAGCAATA
	mMsk2 MUT	TCAATGGGCGGGGGTCGTT	-
		**Target Site Protector**	
	VEGF TSP	TCCGGACCCAAAGTGCTCTG	
	NT TSP	GTGTAACACGTCTATACGCCCA	

Supplementary Table 2: Oligonucleotide Sequences and TaqMan Assay IDs
